# Becoming more integrated into the community: a qualitative study of learners’ experiences of the learning environment in a longitudinal integrated clerkship

**DOI:** 10.3389/fmed.2025.1609051

**Published:** 2025-07-11

**Authors:** Martina Kelly, Grace Perez, Rithesh Ram, Nicolle Begert, Anil Keshvara, Aaron Johnston

**Affiliations:** ^1^Department of Family Medicine, Cumming School of Medicine, University of Calgary, Calgary, AB, Canada; ^2^Distributed Learning and Rural Initiatives, Cumming School of Medicine, University of Calgary, Calgary, AB, Canada; ^3^Riverside Medical, Drumheller, AB, Canada; ^4^Department of Emergency Medicine, Cumming School of Medicine, University of Calgary, Calgary, AB, Canada

**Keywords:** undergraduate medical education, rural curriculum, continuity, learning environment, integrated community clerkship, LIC, Qualitative, distributed learning

## Abstract

**Background:**

While the significance of continuity in the learning environment of longitudinal integrated clerkships (LIC) is widely acknowledged, most studies have focused on continuity of the learner-preceptor relationship and learner-patient relationship. Yet learning environments contain a myriad of wider social dimensions, such as personal relationships, interactions with members of the multidisciplinary team and the broader social context of rural communities; learning is situated within a broad social system. This study aimed to understand how learners experience learning during a LIC.

**Methods:**

Qualitative interviews involving learners in a final year LIC in Western Canada were analyzed inductively, informed by Bronfenbrenner’s ecological systems theory and team reflexivity.

**Results:**

Of the LIC cohort of 22, 18 consented to be interviewed. The participants were mature, had previous careers and most had families with them. Beyond the continuity of relationships with preceptors and patients, the study uncovered other factors that influenced the learning of the LIC students. Apart from students’ interactions in the clinical settings (patients, preceptors and other multi-disciplinary teams), factors such as personal relationships, community connections, learning in a resource-strained environment, geographical isolation, and other socio-political dynamics, impacted the LIC learner experiences of continuity and community integration. The results showed that LIC students were self-directed in their learning and the LIC experience shaped their professional development and facilitated their readiness for future residency.

**Conclusion:**

The relationship between learner and primary preceptor is central but the overall experience of the learning environment is much broader and more complex. Much of the richness of the LIC experience is embedded in the complexity of the learning environment. The use of Bronfenbrenner’s ecological systems theory as a framework for understanding the complexity of the learning environment will be of interest to LIC leaders. The authors recommend potential action points at multiple system levels for medical schools to support the experiences of continuity and integration in the LIC environment and enhance students’ professional journey. These will also provide supports for the ongoing active advocacy work regarding achieving a sustainable rural health workforce now and into the future.

## 1 Introduction

Continuity is a foundational concept in learning within longitudinal integrated clerkships (LIC) (1). While continuity was initially defined in relation to the duration of LIC placements (2), later literature identifies several types of continuity in LICs: preceptor continuity, patient continuity, and ‘contextual continuity,’ which refers to continuity of space and place (3). The value proposition underpinning continuity is the opportunity for learners to develop relational continuity that supports learning and identity formation through immersive experiences in a continuous learning environment. To date, the concept of continuity of learning in LICs has focused primarily on learner-preceptor or learner-preceptor-patient relationships, with several recommendations for creating learning environments that support continuity for preceptors and students (1). Nevertheless, LIC learning environments are complex and often extend beyond these dyadic (or triadic) relationships and include continuity experiences with local hospitals, healthcare teams, and community organizations. Moreover, LICs are situated within a broader medical education environment that encompasses political and social contexts (4–6), which (in)directly influence medical students, their learning experiences, and career choices. Government policy, healthcare funding models, and rural health workforce are examples of contextual factors outside of traditional educational considerations that have substantial impact on the LIC learning environment. Education in longitudinal integrated clerkships can then be viewed as a social process, with learning and learners situated in complex and often nested learning environments.

The importance of the learning environment and its impact on learning is well established in medical education. The term environment refers to ‘that which is around or surrounds’—encompassing the physical and social aspects of a particular setting. It describes the common ways in which people interact with one another, the tone of the social and cultural climate, as well as the organizational structures and physical surroundings that influence learning (7). This broad perspective of a learning environment underscores the importance of examining learning beyond interactions between the learner, preceptor and/or patient (for example, in the consultation room) to consider ‘all that surrounds’ learning. A key feature of learning environments is their dynamic nature, shaped by the people and working relationships that emerge from underlying patterns of patients, locations, practice, education, and society, and from the unpredictable interactions between these patterns (8). One way to study the complex interactions of learning environments is through systems theory (9, 10). Systems theory is a multidisciplinary framework that examines how complex systems function by analyzing the interactions and relationships between various components (11).

**Figure 1:**
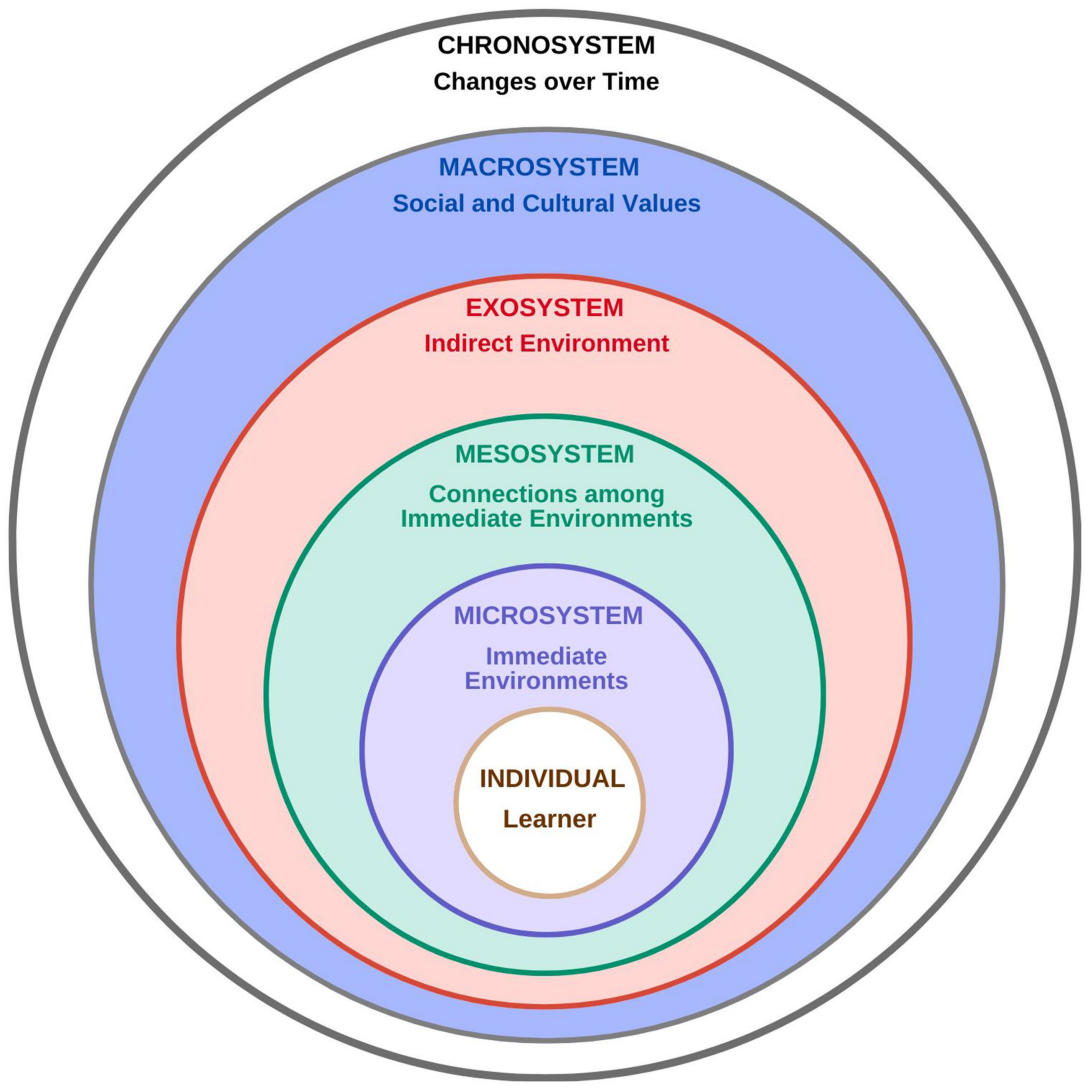
A diagrammatic representation of Bronfenbrenner’s ecological systems theory.

One of the most applied systems theories is Bronfenbrenner’s ecological theory (12–15). Originally developed to comprehend human development, Bronfenbrenner’s theory has been used to understand student learning and the educational environment across various training contexts (16, 17), including medicine (18, 19) and rural learning (20). Bronfenbrenner proposed that the overall environment consists of several layers or sub-environments, each nested within one another, with each layer exerting its own impact on the individual (Table 1, Figure 1). Conceptualized in this manner, all the sub-environments where learning and training occur are interdependent. A strength of the model is its focus on the lived experience of the learner in context, highlighting the bidirectional and dynamic interactions between an individual (the learner) and their environmental sub-systems (16).

**Table 1:** Bronfenbrenner’s ecological system levels.

System level	Explanation
Microsystem	The microsystem is the innermost layer and consists of the immediate settings of the learner, including the interpersonal relations and settings in which the individual lives. A “setting” in the microsystem is defined as “a place where people can readily engage in face-to-face interaction” Bronfenbrenner [(13), p. 22].
Mesosystem	The mesosystem, comprises the linkages and processes taking place between two or more settings containing the learner (micro-systems) i.e. “system of microsystems.” Bronfenbrenner described 4 types of interaction: multi-setting participation (working in different settings), inter-setting communication and inter-setting knowledge (how communication and knowledge is shared (or not) across settings) and indirect linkage (interactions that do not involve the learner but impact their experience).
Exosystem	The exosystem describes events that do not directly involve a learner but still have an impact on them.
Macrosystem	The macrosystem includes overarching cultural values, economic systems, political ideologies that shape the broader environment in which the learner is studying.
Chronosystem	This system represents the dimension of time, considering how both personal and societal changes over time influence a learner’s development.

While the broader context of Longitudinal Integrated Clerkships (LIC) is widely discussed in commentaries and recommendations for practice, the broad context, to the best of the research team’s knowledge, has received less attention from the learner’s perspective. On one hand, this is understandable given the importance of the preceptor-learner relationship as a source of continuity in LIC learning. However, it also overlooks the layered and often complex additional relationships that learners encounter – relationships that can either support or undermine learning. Furthermore, LICs are frequently embedded in socio-political contexts, driven by medical schools’ and funders’ commitment to developing tomorrow’s rural workforce, yet they often operate with fragile funding and limited community resources (21). At the same time, they are often evaluated on par with more well-resourced central university hospital placements. In this study, a LIC program has been established for 15 years, during which the context of LIC learning has evolved. Specifically, rural communities in the authors’ province are experiencing increasing shortages of physicians and healthcare professionals, becoming a focal point for community advocacy and broader political negotiations.

The pedagogical soundness of the LIC model is well established (22–28) and overall outcomes of LIC connect favorably to desired workforce outcomes in primary care and rural settings (29–32) However, the mechanism of the connection between the LIC model and workforce outcomes is not fully understood. With this in mind, the authors sought to explore the totality of the learner experience, and to examine the learning environment beyond the preceptor-learner relationship. Developing a more holistic framework of the LIC learning experience will inform LIC leaders furthering the development and refinement of LIC placements.

## 2 Methods

### 2.1 Setting and participants

At the authors’ institution, the rural longitudinal integrated community clerkship (LIC) program is designed for students in their final year of Undergraduate Medical Education (UME) training. This program immerses students in a rural or regional community for up to 8 months, where they learn rural generalist medicine and various specialties in an integrated manner. It has been operating for over 15 years. Students apply to the program using a formal application process early in the second year of the three-year accelerated medical curriculum. Selection includes both an application and structured interview, which have been iteratively developed by the LIC committee to focus on student learning goals, rurality, and readiness to work in a self-directed learning environment. Neither academic marks nor class standing are considered for selection. Typically, there are more LIC applicants than positions, so all eligible students who apply are interviewed. Students who are not selected for LIC enter the traditional rotation-based clerkship (RBC). A two-person interview panel uses structured questions. Students are chosen based on an evaluation of their written applications and interviews.

The rural and regional communities selected for the LIC are located in central and southern Alberta, and one is in the Northwest Territories, ranging in size from 2,700 to 99,000 in population (Table 2). In line with the definition of rural by Statistics Canada (33) and Warren (34), all communities but one are classified as rural (population <30,000), and all but one are more than 80 km from an urban center with a population greater than 50,000, with one community labelled as a remote location (35–37). The factors required for a community to become an LIC site include (i) interested preceptors who are in long-term practice in the community, (ii) adequate physical space in clinical settings to host learners, (iii) sufficient breadth of community and hospital-based practice to meet the clerkship curriculum learning objectives, (iv) availability of housing for LIC students embedded in community. Students have a primary preceptor and work with various other physicians in the community to gain a breadth of experience across rural generalist medicine. Students follow patients through many care settings, including patient medical home primary care clinics, emergency departments, hospital inpatient wards, operating rooms, delivery rooms, continuing care facilities, and patients’ homes, working with a range of preceptors and multidisciplinary team members. The longitudinal aspect of the LIC enables students to appreciate the natural history of illness, grasp the significance of continuity of care, and adopt a patient-centered approach within the supportive framework of a rural healthcare team. Preceptors are primarily family physicians with rural generalists, many possessing enhanced skills certification in the focused clinical areas, including mental health and addiction medicine, care of elderly, emergency medicine, enhanced surgical skills, family practice anesthesia, obstetrics surgical skills, palliative care and sports and exercise medicine (38). The LIC provides the necessary learning environment for students to achieve the objectives for the final year of the MD program. Medical students from the LIC cohort of the graduating class of 2024 (*n* = 22), who were distributed across 13 rural communities, were recruited to participate in the research study.

**Table 2:** Description of the rural communities and distribution of LIC student placements.

Community	Distance* (Km)	Population (approx.)	Start year	Physicians	Students (Class 2024)
Brooks	190	14,451	2013	24	1
Canmore	104	14,000	2009	66	2
Cardston	234	3,900	2016	9	2
Drumheller	135	8,100	2008	19	2
High River	67	13,600	2008	39	2
Lethbridge	212	99,000	2015	>150	2
Pincher Creek	217	3,600	2008	7	2
Raymond	247	4,199	2023	10	1
Rocky Mountain House	215	7,000	2010	23	2
Stettler	227	6,000	2019	15	1
Sundre	116	2,700	2008	14	1
Taber	263	8,400	2008	14	2
Yellowknife, NWT	1,748	19,600	2011	35–40	2

* Distance from Calgary, AB.

### 2.2 Study design

This exploratory qualitative study is informed by a social constructivist epistemology that recognizes learning as a socially constructed process within various contexts (39). Social constructivism was appropriate for the research question as it emphasizes that knowledge and meaning are constructed through interactions with others and the sociocultural environment. It allowed the authors to explore how learners construct meaning from their specific rural context(s) based on their individual educational experiences (40). The authors opted for a qualitative methodology to foster deeper engagement with participants, allowing them to share their experiences openly rather than limiting their responses to predefined questions typical of an end-of-placement survey. Ethical approval was granted by the Conjoint Health Research Ethics Board (CHREB) of the University of Calgary.

### 2.3 Data collection

The authors conducted semi-structured interviews to explore student learning experiences during the LIC. The interview guide was developed over several meetings of the study team. Development was informed by historical exit interviews performed by the LIC program and modified and expanded to probe into different aspects and layers of the LIC experience. The interview guide included questions about aspects of the participants’ medical training and rural immersion experiences (see Supplementary Appendix 1). One of the co-authors (NB) conducted the interviews via Zoom in May 2024. To ensure a conversational tone, the interview guide was applied flexibly. Interviews lasted between 45 and 60 min. They were recorded and transcribed verbatim by a professional transcription service, which removed any identifiers. As only one to two learners are assigned to each site, interactions and experiences described could be identifiable despite efforts at anonymization. For this reason, to protect participant confidentiality, individual interview transcripts are not publicly available.

### 2.4 Study team’s reflexivity and positionality

The research team consisted of three physicians experienced in educational leadership (RR, AJ, MK), two research staff adept in qualitative research (GP, NB), and a former program learner (AK). RR and AJ serve as academic leads for the LIC program. AJ is the associate dean for distributed teaching at the medical school, overseeing all distributed teaching activities, while MK previously held the position of family medicine clerkship director. RR also directs the LIC program, works as a rural generalist physician, and acts as a clerkship preceptor. He reviews all student applications and makes decisions regarding student placements. NB serves as the community placement coordinator for the LIC program and directly addresses student concerns throughout their clerkship immersion. GP supports the various scholarship activities of the distributed teaching faculty, preceptors, and medical learners. AK is a practicing family physician from a rural community and a postgraduate learner in an emergency medicine enhanced skills program.

Reflexive thematic analysis (RTA) was used as the primary approach in this study. RTA is an approach for interpreting data patterns, themes, and meanings, specifically informed by researcher reflexivity. It relies on researchers critically reflecting on their values and assumptions throughout the research process. This was particularly significant for this study, given the involvement of academic leadership in the LIC program. On one hand, the participation of RR and AJ provided rich contextual detail, but their leadership roles risked emphasizing positive experiences while downplaying less favorable learner experiences. To support their reflexivity, the authors periodically paused to examine their accounts using reflexivity questions, as outlined by Crabtree and Miller (41). The team balanced their closeness to the data by being intentionally attentive to negative comments and counter themes in the data. Team reflexivity was further reinforced by GP and MK’s more distant relationship with the program.

### 2.5 Data analysis

Data analysis involved two iterations. In the first phase of analysis, the authors openly coded the data using reflexive thematic analysis (RTA) (42). Analysis commenced with team members reviewing the transcripts broadly to familiarize themselves with the data and consider patterns and preliminary themes. Each interview transcript was read by at least 2 members of the research team. This was followed by an open coding phase, during which each team member independently examined the transcripts, made remarks, highlighted significant quotations, and engaged with the data based on the diverse experiences as physicians, educational leaders, and preceptors involved in the LIC program. Coding for this project was done manually. Subsequently, the authors gathered to discuss the initial codes. Each interview was then coded by two members of the research team, who reconvened to refine the coding scheme. Through a series of meetings, the authors deliberated and enhanced the coding while reflecting on the various stakeholders that influenced (or hindered) a learner’s experience, as well as the extent to which this affected each participant’s overall learning journey.

A ‘thinking with the theory approach’ was used to iteratively deepen understanding between the data and the framework (43–46). As the analysis developed, the authors drew on Bronfenbrenner’s ecological systems theory (13) as a sensitizing theoretical framework to enhance the emerging interpretation. Team members reread the codes, using the conceptual map to identify the dynamics among varying stakeholders and their level of influence. To ensure the interpretations were grounded in the data, each team member revisited a subset of interviews to check the codes against the overall model, confirming that the coding labels aligned with the research team’s thoughts and interpretations. This conceptual framework was employed to map codes and identify themes, demonstrating connections between issues and experiences identified by the participants.

For example, preliminary reading identified the learner-preceptor relationship as being of central importance. Subsequent deeper reading, with the model in mind (i.e., thinking with the theory approach) uncovered the importance of day-to-day relationships with other health professionals and alternate preceptors. Through systematic analytic engagement with the raw data, acknowledging the team’s personal positioning and engagement with theory, the authors constructed the final interpretive synthesis.

## 3 Results

### 3.1 Demographic profile

Of the 22 students in the LIC program, 18 (82%) consented to be interviewed. The average age was 30.8 years. Most of the students were female (11/18, 61%) and had partners (13/18, 72%). Among the partnered students, 4 (22%) had children. Two-thirds of the students had a rural background (12/18, 67%). Almost three-quarters expressed interest in Family Medicine at the outset (13/18, 72%) and the same number of students eventually matched to a Family Medicine Residency program after LIC (13/18, 72%), mostly in a rural or remote setting (9/18, 50%). (Table 3).

**Table 3:** Demographic profile of participants.

Demographics		N	%
LIC cohort size		22	
Participated in the study		18	100
Gender	Male	7	39
	Female	11	61
	Other	0	0
Average age (at entry to LIC)		30.8 years	
Graduated rural high school	Yes	10	56
	No	8	44
Family status	Single, no children	5	28
	Single with children	0	0
	Partnered, no children	9	50
	Partnered with children	4	22
Self-reported rural background	Yes	12	67
	No	5	28
	No data	1	6
Career interest	Family Medicine	11	61
	FM, Focused Practice	2	11
	Anesthesiology	2	11
	Emergency Medicine	1	6
	Internal Medicine	1	6
	Undecided	1	6
Previous professional background	Advising/consultancy	3	17
	EMT/paramedic	1	6
	Physician assistant	1	6
	Physiotherapist	1	6
	Psychologist/mental health/social work	4	22
	Registered nurse	5	28
	Research and policy	3	17
Residency match after LIC	Family medicine - Rural	8	44
	Family medicine - Remote	1	6
	Family medicine - Urban	3	17
	Family medicine and emergency medicine	1	6
	Anesthesiology	2	11
	Diagnostic radiology	1	6
	Emergency medicine	1	6
	Internal medicine	1	6

### 3.2 Qualitative interpretation

**Figure 2:**
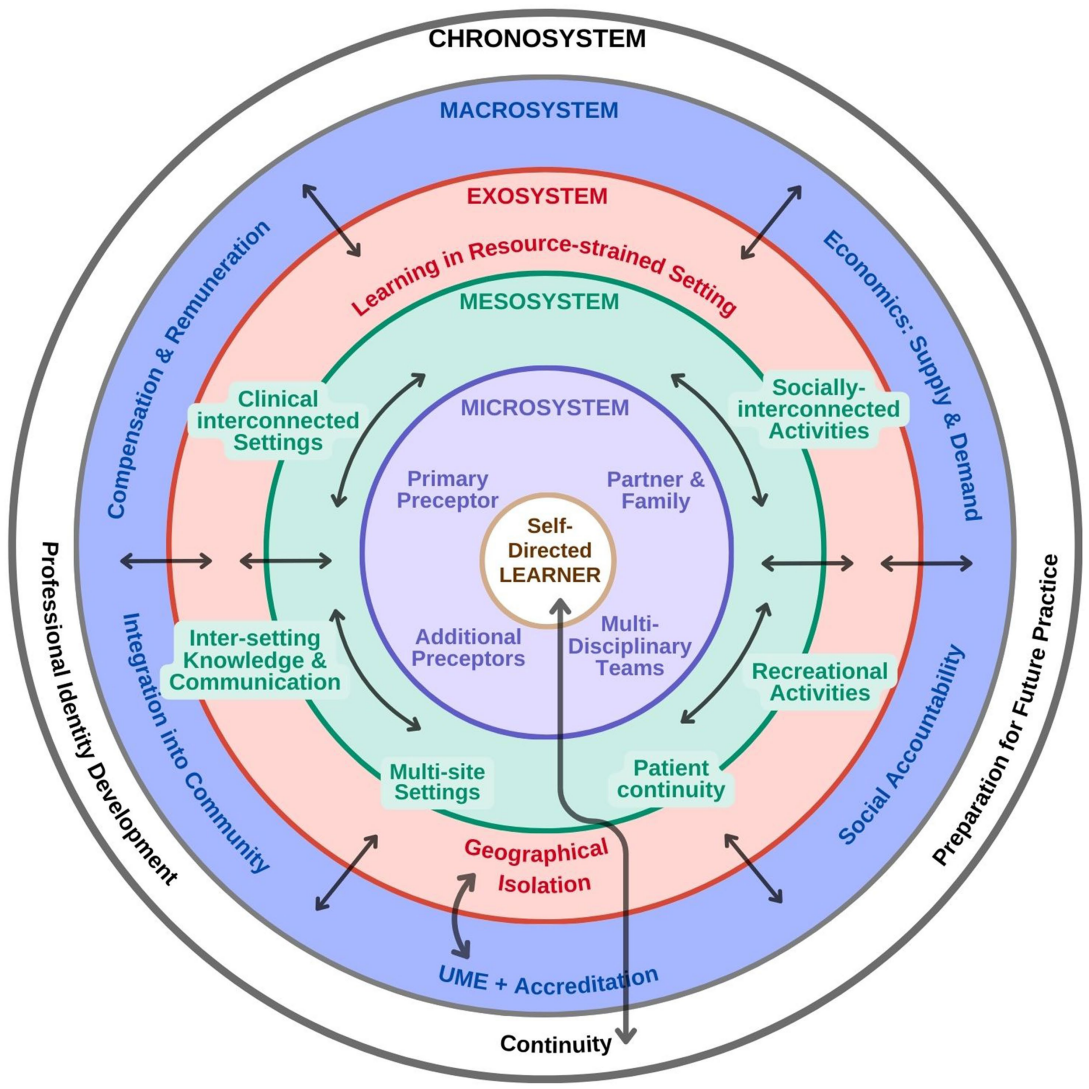
Dynamic model of LIC learning environment.

The final interpretation of the data is represented in Table 4 and Figure 2. Table 4 outlines the domains of Bronfenbrenner’s theory to illustrate the range of stakeholders and interactions that contribute to how learners experience the learning environment in a longitudinal integrated clerkship. These are represented in Figure 2, adding arrows and directional symbols to help readers appreciate the dynamic (and individual) nature of how the various components of the learning environment can support or detract from a learner’s experience.

**Table 4:** LIC learning environment: sub-system content examples and codes, considered through Bronfenbrenner’s ecological theory.

Bronfenbrenner’s system level	Content	Codes
Learner	The self-directed learner is at the center of the learning environment.	Participants identified as motivated, self-directed learners. They described actively seeking out learning opportunities. They especially valued experiences that offered hands-on learning.
Microsystem (immediate environment)	The immediate environment of the learner comprises of relationships and interactions with: Primary preceptor Additional preceptors Members of multidisciplinary team Personal relationships	Preceptor-learner relationship: preceptors regarded the learner as a professional, and a person. Learners valued the additional mentorship provided by other preceptors. Learning was enhanced by multidisciplinary teamwork. Learners’ interpersonal relationships were integral in their learning.
Mesosystem (linkages and relationships between systems)	Factors include the direct experiences with peers, preceptors, patients in the learning environment and experiences of the curriculum: Working in multiple settings Social activities with preceptor and local team members Social activities in the community Exposure to patient continuity Indirect linkages with Undergraduate Medical program	The LIC allowed multi-setting participation among learners. The LIC promoted inter-setting knowledge and communication. The learners appreciated being seen and felt welcomed, promoting a sense of commitment to community. Participant learning was enhanced by exposure to patient continuity. Participants experienced indirect linkages that impacted their learning.
Exosystem (indirect environment)	These factors may include: Lack of clinical resources Geographical isolation	LIC training in a resource-constrained environments impacted the participants’ learning. The geographical isolation indirectly impacted learning. Learners witnessed burnout among rural healthcare professionals.
Macrosystem (social and cultural factors)	These factors exist outside the medical learning environment (beyond the medical school or clinical learning environment), but that influence the inner “sublevels” and the learner. Accreditation requirements Economics of supply and demand Political system – models of compensation and professional remuneration Social accountability	In addition to the academic requirements of the medical program, the LIC learners acquired deeper understanding and awareness of the geopolitical, socioeconomic issues affecting rural Canada, which in turn impacted their sense of social accountability.
Chronosystem (changes over time)	These are changes related to personal and professional conditions and the learning environment associated with time. Professional identity formation Preparation for future practice	The LIC program increased participants’ clinical confidence and preparedness for next level of training.

#### 3.2.1 The learner

Analysis of the transcripts indicated that the learner and their approach to learning is central to how learning is subsequently experienced. The sub-themes noted are (i) that participants identified as motivated and self-directed learners, (ii) that learners were actively seeking learning opportunities.

Participants compared their LIC experience to their colleagues’ experiences in traditional block-based rotations and indicated that the LIC had less formal learning than traditional block-based rotations, but significantly more opportunity for experiential, hands on, learning. Participants felt capable of navigating resources to retrieve formal information for self-consumption, while highly valuing for direct hands-on experiences, with supervision and feedback by their preceptor.

Multiple participants identified strategies they used to seek out learning opportunities, including seeking out specific experiences with specific preceptors, willingness to be being pushed beyond their comfort zones in the context of positive preceptor relationship, recognition of the limits of their own abilities, and active thinking about taking on progressive levels of responsibility over time. This approach to learning connected to the microsystems level as an enabler of the learner-preceptor relationship, particularly around mutual trust and mentorship.

*P2: Those of us who decide to pursue LIC are probably, on balance, people that aren’t necessarily afraid of undifferentiated situations or the new and the unfamiliar. …Being successful in LIC definitely requires initiative and a self-directed approach to training. And kind of managing one’s time…… There’s going to be a ton on the exam that I did not see in clinic and that’s up to me to learn independently regardless.*

#### 3.2.2 The microsystem

The microsystem refers to the environment directly surrounding the learner, including their immediate settings and interpersonal relationships. In addition to the immediate relationship with their primary preceptor, participants described the value of working with additional preceptors, locums, and multidisciplinary team members. They also emphasized the role of personal relationships, such as with partners and family. These relationships supported or undermined experiences of continuity.

##### 3.2.2.1 The preceptor-learner relationship related to knowing the learner as professional, and a person

Learners most significant interaction was with their primary preceptor. Most participants described a collaborative and trusting relationship with their main preceptor, which developed over time. They explained how the relationship grew as the preceptor and learner became familiar. Preceptors dedicated time to understanding learners’ learning approaches, interests, and expectations, tailoring learning objectives accordingly. They also got to know their learners as individuals, exploring their personal interests (e.g., hobbies, career aspirations) and supporting their personal development as they adjusted to life in rural communities. This fostered a safe learning environment where learners felt comfortable asking questions. They also appreciated how their preceptor granted them increasing independence, trusting them as their skills developed and challenging them at times, scaffolding their learning to expand their ‘comfort zones’.

*P9: Because he got to know me very well, he was very good at knowing what my limits were, and then kind of pushing me to my limits, or pushing me a bit outside my comfort zone. But he was always there.*

The learner-preceptor relationship was described as ‘*respectful, engaged, very supportive’* (P4). Learners valued the personal mentorship offered by their preceptors, with opportunities to discuss career choices, navigate professional boundaries, and experience living in a rural community.

*P5: Yeah, no, I think he’s definitely a role model. My preceptors all really balance family and work very well. So, in a way, they were very dedicated to their profession, but they were also very dedicated as parents and as community members. And I thought that they were role models in the sense that they had, they really had a serving attitude towards their patients, and they really had that kindness towards their patients. But at the end of the day, they also said, “I’m going to go home,” or “I’m going to go see my child’s basketball game,” or “We’re going to go see our grandkid,” or something. So, to me, that was a really, that is a role model to me.*

However, not all participants experienced supportive relationships. One participant described challenges in initial relationship formation with a preceptor who was absent at the beginning of the LIC due to a personal emergency. Another participant described the challenge of working with a preceptor with different world views in specific areas of practice such as harm reduction, and the resulting need to reflect and broaden their work with other preceptors.

Engaging with locum doctors was identified as a particular challenge. Learners in communities with high numbers of locum physicians faced challenges in educational continuity, progressive autonomy and even in securing learning opportunities when some locums declined to work with LIC learners.

*P1: The biggest challenge I faced … was that there is no continuity in terms of the people who are working there. Everybody’s a locum. And so, when you have a new preceptor that does not know where you are at in learning, then they go, “You’re a medical student, so I’m going to assume you do not, you are at a certain level… Whereas every time I was there, there was someone brand new who’s, ‘Ooh, I do not know if I’m going to let you do anything today.’” And for me, that was the biggest struggle.*

##### 3.2.2.2 Participants valued working with the other preceptors that provided additional mentorship

As they rotated through community hospital settings and worked with other physicians in the area, learners remarked on opportunities to observe how different preceptors approached clinical care and experienced a form of distributed preceptorship, where each community member actively supported their learning.

*P2: So overall, the relationships that I had with all of the preceptors I ultimately did have was very positive. I felt supported and valued, and that my development as both a physician and as a person were prioritized……. my development as a physician ……was something that they were invested in…… They also….took a personal interest in my growth and development. And that made me trust in their methods and in their, kind of their mindset when it came to approaching educating me, which was wonderful.*

##### 3.2.2.3 Participants said their learning was enhanced by multidisciplinary teamwork

Alongside collaborating with other physicians, participants highlighted the importance of engaging with and learning from other multidisciplinary team members (27), notably when they witnessed its impact on patient care.

*P17: I think it gives you the opportunity to have stronger relationships with those allied health professionals, because they, most of them have permanent lines, the dieticians and the social workers and the nurses……I know that I can safely discharge patients when I’m in the rural location, because I’ve had the opportunity to have them assessed properly, and come up with appropriate discharge planning, and homecare planning, to get them home. And because you have those relationships, our inpatient homecare navigator has a direct relationship with the community homecare planner, and so those things happen a little bit more efficiently. But I think that just shows relationship actually means something. Especially, you are working in a small group of people that can kind of share their own approach to ethics and decision making, which is great.*

##### 3.2.2.4 The participants’ interpersonal relationships were integral in their learning experience

Participants emphasized the prominence of maintaining their social relationships, particularly with partners and children. This included, for example, travelling back and forth between the community and city to see partners or their children if enrolled in city schools. Access to housing to facilitate partners and/or family living with them or visiting significantly impacted their experiences.

*P5: For me also, again I went with my family, with my fiancé, and so that I would say contributed really. A big core portion of my success was, so I think it’s really good that LIC allowed for children and for spouses and for family members to come with them. I think that really contributes to the learner success… Having the housing also contributed to the success, knowing that I do not have to find a rental place. The move can be quite quick, it’s a quick turnaround time to get from the orientation to your site.*

#### 3.2.3 The mesosystem

The mesosystem acknowledges that factors within the microsystem are not isolated but interlinked. It refers to the connections, relationships, and linkages between various aspects of the microsystem - in other words, how the different spheres of influence intersect.

##### 3.2.3.1 The LIC allowed multi-setting participation among learners

Participants shared experiences in various settings, from their preceptor’s clinic to local hospitals, including the emergency department, obstetrics, surgery, and long-term care. Engaging with patients in diverse contexts deepened learners’ understanding of the importance of continuity of care, especially for patients. Observing patients in the emergency room and later in community settings reinforced this concept. Additionally, several participants highlighted the value of working with pregnant patients and accompanying their journeys alongside their families throughout their LIC.

*P10: I saw patients in prenatal clinic, and then I saw them in emerg, and then I saw their husbands in emerg. And then I got to deliver their baby with them, and then I was like. I had great relationships with the patient and her husband, and now their new baby, and then I saw them in family clinic, it was just great.*

##### 3.2.3.2 The LIC promoted inter-setting knowledge and communication

As learners moved between settings, they reflected on how health professionals’ knowledge of the patient, their context, and the local healthcare infrastructure promoted personalized, flexible care, which one participant described as ‘*this is how we do things here’*.

*P18: Because they knew us by name and who we were, they were good about discussing things and coming up with collaborative plans together rather than bouncing notes back and forth. So, I think it, it really, it’s crucial I think, especially when you are working in rural settings, to have everybody with that same mindset of engaging in that collaboration……These are your people; this is your community. You, as a rural generalist, have an active interest in the health of the people you look after because they are also the people that you see at PTA meetings or across the hockey pond with your kids.*

Data also highlight the central role of interconnected knowledge and communication, learned through social (non-clinical) interactions within the micro-system. Opportunities to engage in social time with preceptors and other team members were vital in helping learners feel integrated into the community. Social integration extended beyond the immediate healthcare team and included making connections by attending local events, such as festivals, and engaging in recreational activities and community life.

*P5: The preceptors would actually invite you over to their house or to a party or a get together. And I found that that was actually a really amazing way of building trust, having a meal with their whole family, and getting to know their kids. And going to a barbecue and all of the doctors would be there. So, I thought that that was, so that would be how I built professional trust, but then also personal trust kind of, really just inviting you into the community.*

##### 3.2.3.3 Participant learning was enhanced by exposure to patient continuity of care

The longitudinal nature of LIC allowed participants to understand natural disease progression, management and prevention and helped them become more patient-centred.

*P5: At my rural site, it’s like, what antibiotics are we doing? What are we going to do with this patient? And so, you really do start to think about patient care from start to finish. … And you really feel the weight of that. … most of the people that you see in the rural practice are undifferentiated, you do have some people with chronic or known health conditions. But especially too, you really do have to think in the rural communities, when you do not have all these imaging modalities, you do not have all these specialist consults or someone who’s there to do ultrasound 24/7. So, it really does force you to go back to basics, and think about your differential when you have an undifferentiated patient. So, LIC was very useful in that.*

##### 3.2.3.4 Participants appreciated being seen and felt welcomed, promoting a sense of commitment to community

Social integration was about more than just ‘having a good time’. It helped learners connect with the community, foster a sense of commitment to that community, and understand what it means to live and work as a rural physician.

*P5: Basically, you take your role quite seriously, because you are not hiding behind anonymity, you are not going to never see this person again. And you might treat their whole family, and so you need to do a good job and you are honoured to do a good job… We did try to integrate into the community and we went garage sale-ing every Saturday, and we went to all the barbecues. And if there was a pancake breakfast in [the village], we went to that and we walked around. And we went to the library, and the grocery store, and yeah, we, and corn days, and all of the different things. So, we really did try to integrate into the community, and we found that that was a really rewarding experience. Especially, like I talked about the anonymity of Calgary, I find that to be quite distressing. And I really do not like to be this anonymous cog person, I really like to be someone who has some skin in the game, who has some stakes. So, I really enjoyed living in my small community.*

Not all participants, however, integrated into the community in which they were learning. Some participants found it difficult to settle in and form connections. For others, family commitments, such as having children attending school or partners living in a different community, resulted in their spending less time in the community.

*P8: I tried hard to find things to do for my first couple of weeks here. I did not really meet anyone. I did not know what to do. And so, then it just became the default to go back to the city on the weekends…… But it would have taken nothing for someone my age to be, do you want to go biking around the town on Wednesday night, and we’ll show you some paths, that took me 6 months to figure out.*

##### 3.2.3.5 Participants experienced indirect linkages partly due to the LIC’S geographical isolation

Indirect linkages refer to interactions that do not involve the learner but impact their experience. One example of indirect linkage was a mismatch between learning in the local community and expectations of the undergraduate medical education program, particularly in relation to curriculum coverage and assessment.

*P19: You’ll get amazing practical experience, but for the exams, it’s still very much tailored to the one size fits all clerkship unfortunately. And you need to pay attention to those objectives.*

#### 3.2.4 The exosystem

This level includes factors that are not directly within the learner’s immediate environment but still influence their experience. Participants reflected on the impact of learning in resource constrained rural environments. They described challenges arising from this, including the need to manage logistics around patient transfer, specialist consultation across long distances, and recognition of health disparities arising from geography. While this was identified as a challenge, some participants who planned to practice rural medicine felt that this was an important window into the realities of rural practice and important learning.

*P18: But it was good to see how you could deliver healthcare in that under-resourced setting, which I do not, I think that’s a misnomer. We were, more than often I saw enough complex, interesting stuff in that setting that we could handle there, that was sufficient, compared to the city. So, I think that was good from a learner perspective, to understand what I, and to reaffirm, I have a vague idea of that, in retrospect, going into rural medicine. And now that we are pursuing that, next or this year, in a couple of months, for real. I have now a firm understanding of that, which is going to help…*

##### 3.2.4.1 Participants had to deal with challenges stemming from geographical isolation

Participants also dealt with challenges stemming from geographic isolation. Living at a distance from family and social connections was identified as a particular challenge. This was exacerbated in settings with poor cellular coverage and internet connectivity, which added an additional barrier to maintaining key relationships. Witnessing the challenges of others in relation to geographic isolating also impacted participants. Witnessing the burnout of other health professionals was particularly impactful. Participants noted how burnout in one person could impact others. For example, how temporary staff could get burned out and leave, but how this also caused burnout in the permanent staff responsible for training repeated waves of temporary workers.

*P1: … A lot of the nurses, a lot of allied health professionals up there [remote town] are really burnt out. And a part of that was because they’d be up there [remote town], and then they would get a locum nurse, and then they train her, and then they, and then she’d leave. And so, they were burnt out, because all they were doing was training these people who did not stay, and then they left.*

#### 3.2.5 The macrosystem

This level includes factors existing outside the medical learning environment (beyond the medical school, university, or clinical learning environment), but that influence the inner “sublevels” of the framework and the learner. This may include the wider context in which the school exists, including social, political, historical, and global, as well as other factors, such as professional regulatory or curricular requirements.

Several participants had selected into the LIC due to their interest in working in rural environments. In addition to commenting on the experience of learning to work in less resourced communities, learners commented in the impact this lack of resources had, at times, on the overall morale of a community. Learners were also very aware of external political factors occurring during the course of their LIC, for example political work on compensation models for rural physicians and developed their own opinions about the potential effectiveness of these models on physician recruitment.

*P5: I think that physicians in rural Alberta are remunerated fairly, I do recognize that there’s an extra bit of stress to the job, not having all these access to specialists, not having access to all the scanners, you think a little bit more during the job. But no one was really ever hurting for money. And I feel like, yeah, it’s tricky, because it’s very hard to get people to come out and work in a rural community, unless they really want to. … it’s really hard to incentivize people with money. It’s like, I say that I want to be incentivized with money, that would be fantastic, if I can get [incentives] in signing stuff. But I recognise that that’s not going to retain people who just are doing it for the money, and who will take that two-year contract, and then they’ll get out of there. Hopefully, by living there for 2 years, they’ll recognise that maybe this is the way to go. But yeah, I do not know, I’m of the opinion where it’s like, yes, I love physician compensation in rural areas, because that’s what I want to do. But I think it has to be, it has to start a little bit earlier. It honestly has to start at the recruitment for med school stages You have to just go and recruit rural people, because that’s the best demographic who will actually go back and do good on these service contracts.*

#### 3.2.6 The chronosystem

The chronosystem considers impacts and changes associated with the passage of time. Continuity was the dominant theme in this area with impacts at multiple levels, across other systems. Preceptor continuity, patient continuity, and community continuity were all highlighted, as important elements of a successful LIC experience. Continuity also had a powerful impact on learners’ professional identity formation, learners reported personal changes, growth, and shifts in perspective over time. Likewise, when elements of continuity were disrupted, the impact on the learners’ was significant (as an anti-theme).

##### 3.2.6.1 Preceptor continuity was integral in the students’ learning

Working with a preceptor over time allowed the development of mutual trust as well as mutual adaptation of teaching and learning styles, and graded independence.

*P11: (We) developed mutual trust by basically being both accountable, so he was accountable for me, I was accountable for him and being accountable for our patients. So, we were both accountable for everyone that we are seeing, which I think is the way it works when you are an actual doctor working in fields, and you are working alongside your. And I think that really – he knew exactly where my learning was at, and could trust me with things that I had clearly demonstrated beforehand. And he, that way he was able to kind of allow, trusted me to be very independent, and that’s how we kind of built the trust up between us, and yeah, it worked really well.*

##### 3.2.6.2 Patient continuity was supported by the nature of the LIC experience

Learners emphasized the value of seeing patients over multiple visits and across multiple settings. Learners provided examples that highlighted multiple visits as a strategy to deal with uncertainty, examples of emerging diagnostic clarity. Learners also provided examples of supporting patients over time through grief, challenging mental health issues, and transition between cancer diagnosis and providing palliative care. We have chosen to describe these instances broadly and not link to specific learner quotes, because the specific nature of these quotes could be identifiable for patient, learner, preceptor, and community.

*P7: I saw somebody who was being worked up for chronic cough and then I saw him at a later time, after using a medication and seeing if it was helpful. Kind of reasoning between asthma and GERD and it seemed to be responsive so more likely GERD. I kind of saw people with consistent depressive symptoms and the progression of that and kind of working with non–responsiveness to medications.*

##### 3.2.6.3 Community continuity subsequently evolved over time as participants felt more integrated

Integration into the community over time allowed learners to understand the role of rural physicians as part of the community. Learners also became involved in community events as they settled into the community over time. Some learners described a process of ‘fitting in’ to a community and progressively becoming involved in the day-to-day life of the community beyond the work setting.

*P2: I actually think that was a real strength of UCLIC, where we get to ingrain, integrate into our communities, and really kind of learn the needs of the communities and the patients that we support. I think that was a real benefit there. Disease prevention, health promotion, same. I think one of the cool things about rural family, is these are your people, this is your community. You as a rural generalists have an active interest in the health of the people you look after, because they are also the people that you see at PTA meetings or across the hockey pond with your kids.*

##### 3.2.6.4 In some instances, participants described experiencing disruption of continuity

Disruption of continuity with the primary preceptor was among the most significant challenges an LIC learner could face. Learners who had challenging relationships with preceptors went out of their way to develop relationships and continuity with other preceptors. A learner who experienced significant challenges with their preceptor described how they developed a preceptor relationship with another local physician who became a surrogate preceptor.

*P6: I think he has a very busy schedule with the residents, but I think he probably went out of his way the most to make a point of making sure that I got time and experience and reference letters and things like that.*

However, even when learners did find alternate preceptors, the disruption of the continuity relationship with the primary preceptor had impacts that could not be overcome.

*P6: But no matter how many good things happen to you, if you have a bad primary preceptor, it’s hard to make up for that.*

Continuity was also disrupted in one community due to wildfire evacuation. An evacuated learner discussed the impact of this, and how UME did not fully appreciate the challenges that this disruption caused.

*P16: I know they wanted to get us back into rotation so quickly when we got here, but that is not what [we] needed, we needed at least 2 or 3 days to be able to just breathe and then have somebody sit down with us and say, “How are you guys doing, what do you think that you are capable of handling, without the threat of you now need to make this up later on.”*

##### 3.2.6.5 The LIC experience reinforced participants’ professional identity formation

Learners used transformative terms to describe the impact of the LIC on professional identity formation, emphasizing the importance of time spent in the LIC space as providing the opportunity for these reflections. Discussions of professional identity formation were integrated with discussion of readiness to progress to the next level as a resident physician, ability to manage uncertainty, and progressive readiness to take on higher levels of responsibility in patient care.

*P2: It’s shone a powerful light on me, for me in terms of what actually mattered to me in my career and in my life. I came out of LIC with a very different perspective on what I wanted to do for my career. And what, how I wanted my work and my life and my, how I wanted those two to interact. And that LIC, I think, in my time in [Rural Community] really solidified, clarified in my mind, a lot of things about what I wanted from my career and my life, that I did not fully appreciate going in. And I feel coming out of it very confident in my residency decision, having experienced those, for a lack of a better word, epiphanies about what I wanted for my life and my career.*

## 4 Discussion

This study used a qualitative data set from a cohort of LIC learners to understand the LIC educational experience from the learners’ perspective. Employing Bronfenbrenner’s ecological systems theory (12–16, 47) as a framework, the study investigated LIC student experiences across multiple systems within the rural LIC community. Table 5 presents a summary of proposed action responses and suggested interventions by system level to further support experiences of continuity and integration and enhance student learning.

**Table 5:** Action points to support experiences of continuity and integration in LICs.

Bronfenbrenner’s system level	Components/Factors	Take-home	Action
Learner	The Learner	Students selected for LIC are often self-directed and relatively independent, valuing opportunities for autonomous hands-on learning	Make this clear to students, i.e., does this align with their approach to learning? Consider a session on self-directed learning, time management, etc. at orientation.
Microsystem (immediate environment)	Primary preceptor relationship	Preceptor selection is key. Preceptors were flexible, tailored learning to the learner. They scaffolded learning to extend comfort zones (zone of proximal development) (52). Preceptors got to know the learner as a person and were engaged in a mentoring relationship.	Carefully select preceptors and support through faculty development. Continuity of preceptorship key, e.g., if planning leave, this should be discussed at central and local level. Supporting open communication, e.g., to enable preceptors and learners to communicate if inter-personal differences to program leadership. Consider faculty development that explicitly outlines idea of scaffolded learning.
	Working with additional preceptors	Students value variation and working with a range of preceptors.	When establishing a LIC placement, it is important for the rest of the clinical team to be aware of their role and how they support (or not) a learner. The role of locums as teachers within LIC requires specific consideration. While these can be valuable learning opportunities, there is considerable variation in locum teaching interest. Consider the development of specific resources that can quickly orient locums to the concept of LIC and the role of the LIC learner for sites using locum physicians.
	Working with other professionals	Same as above	Same as above
	Personal relationships	Family and friends play a supportive or undermining role.	Make clear to learners how personal relationships are /are not supported through the program, e.g., housing, access to community recreation activities (see below).
Mesosystem (linkages and relationships between systems)	Working multiple settings	Working in different contexts helps learners understand how information and knowledge ‘flows’ across settings to support ‘contextual continuities’.	Ask learners to write a reflection on how working in different contexts in a local community helps foster their understanding of generalist practice.
	Exposure to patient continuity	Continuity of care is fostered by learners working in different settings.	Help students care for a cohort of obstetrical patients and patients with mental health issues can support their understanding of continuity of care.
	Social activities with preceptor and local team members	Social activities play a fundamental role in fostering student integration.	Make explicit to preceptors and teams. Consider ‘hosting’ /funding a ‘welcome’ event at the orientation of the student to the community.
	Community connections: Role of partner/family Recreational activities	Partner and family help integrate the learner and foster connection to the community	Have a ‘recreation’ activity list/engage a community member to help facilitate getting to know local resources and activities. Consider local gym/recreation pass.
Exosystem (indirect environment)	Factors may include: Lack of clinical resources Geographical isolation	Students learn in resource-strained environment Students felt disconnected with UME during their placement due to the geographic distance	Incorporate the physician advocacy role as an explicit learning objective relating to the rural healthcare environment. Ensure that students have clear ways to connect with UME and urban campus resources.
Macrosystem (social and cultural factors)	Socio-cultural factors: Accreditation requirements Supply and demand Models of compensation Professional remuneration Social accountability	LIC students acquired deeper understanding and awareness of the geopolitical, socioeconomic issues affecting rural Canada, which in turn impacted their sense of social accountability.	Preceptors should include these issues in teaching and conversation. Ensure that the connection between the LIC learning environment and social accountability is clearly reflected as a curriculum objective.
Chronosystem (changes over time)	Changes related to personal and professional conditions over time: Professional identity formation Preparation for future practice	Teaching and supervision will change over the course of the LIC to reflect developing professional identity and capacity for independence.	Support preceptors through faculty development, to adapt their approach to teaching and supervision over time with LIC learners, thereby supporting professional identity formation and readiness to transition to postgraduate training.

While much of the existing literature on the LIC learning environment has concentrated on the learner-preceptor relationship (48–51), the findings of this study uncovered a previously recognized but poorly acknowledged level of complexity within the LIC learner environment. Although the learner-preceptor relationship was undoubtedly significant, the learning environment, as perceived by the learners, was layered and nuanced, encompassing experiences across various levels of Bronfenbrenner’s framework. The learner-preceptor relationship does not solely define the learner experience; rather, the learner’s experience of integration across the layers of the framework throughout their journey ultimately determined the quality of their experience.

The demographics of the learner group and their self-descriptions revealed a mature cohort, many of whom have prior professional experience before entering medical school. Their self-descriptions indicated that they are self-directed and prepared to take on a challenge. In the context of this subgroup of the overall UME class, who self-selected into a rural LIC, it is important to consider that this self-perception may not represent all medical students and that the process of choosing the LIC may select for such personal characteristics. As mature students, many with prior professional backgrounds, individuals may have also had different experiences of key elements of clerkship, including ability to act as a self-directed learner, professional identity formation, and integration into healthcare teams. Although this LIC cohort was typical for our medical school, some caution is warranted in generalizing these results to all learners without regard for their individual backgrounds.

At the microsystem level, the relationship between the learner and the primary preceptor remains central to the overall learning environment experience. Learners’ descriptions of this relationship indicate that it evolves over time within the LIC. Various types of relationships were described, including mentorship, coaching, senior–junior colleague interactions, and role modelling. Across a range of relationship constructs, the boundaries and nature of the relationship are framed in terms of professionalism. This suggested that there was not a single successful or desirable learner-preceptor relationship type; rather, it likely evolved to meet the specific needs of both the learner and the preceptor. Furthermore, the data highlighted the value students placed on working with a range of preceptors, which we referred to as a type of ‘distributed preceptorship’ across a community setting. Historically focus has been on fostering the learner-preceptor relationship, but consideration should also be given to preparing the broader healthcare team to successfully work with LIC learners.

Learners with challenges in the learner preceptor relationship described impacts beyond the relationship itself. One learner, whose primary preceptor was unavoidably absent at the start of the LIC described challenges integrating into the health system and community. Experiencing preceptor discontinuity also made it difficult for learners to gain progressive independence, describing starting from scratch with new preceptors. Some learners experienced philosophical differences with preceptors, which led to introspection and consideration of how they would personally practice in the future.

The microsystem also includes a range of other physicians and health professionals who the learners worked with and alongside. This aspect of the LIC experience was generally positively described as an opportunity to see a broader range of practice and learners generally felt welcomed in these experiences. However, a particular challenge in working with locum physicians was noted, including instances where locums preferred to not work with a learner. Since locums are typically less connected to a community this could represent disinterest in teaching an LIC learner, discomfort with the teaching role, or even unfamiliarity with the concept of LIC. While experiences with locums could be positive, they may be seen as higher risk from the perspective of the learner, who could risk rejection in asking to work with them. Many rural sites are dependent on locums for delivery of clinical services and contact with locums may be unavoidable in the LIC context. We identify this as a specific area to address, including the development of specific resources that can quickly orient locum physicians to the LIC concept, and the role of the LIC learner.

The learners’ experience of the learning environment also involved factors beyond the immediate healthcare environment. Maintaining connections to partner, family, and children was an important factor in the overall experience, as was being welcomed into the community in a way that allowed participation in elements of community life. This interconnectivity describes the mesosystem of the LIC, in which these factors are linked together as part of the total learning environment. For instance, learners discussed the importance of housing options that could accommodate family, and integration into community life. Although such factors may be beyond what medical schools traditionally consider in terms of student placements, they were clearly understood by learners as part of a holistic and integrated learning environment. These interpersonal relationships and community connections were integral to the learner’s experience.

Concepts of continuity also emerged in the mesosystem. Learners appreciated working across different environments such as clinic and hospital and following patients across multiple settings. Communication and care were enabled by both continuity with patients, and continuity between the learners, preceptors and other healthcare workers, where the learner was a known part of the team, and therefore part of team communications. Broader engagement of communities and municipalities may be a beneficial action to foster connection between LIC learners and the activities and amenities that exist within those communities.

An interesting dissonance arose in examination of the position of UME within the model. Elements of UME such as learning goals and examinations were very much part of the day-to-day life of learners as part of the microsystem, but at the same time students felt distant to UME, and that the UME expectations and learning goals were not always aligned with the day-to-day work in the LIC. Learners indicated a disconnect between the content of their day-to-day learning experiences and the expectations of their examinations. Although learners understood the goals of UME training to be generalist in nature, they also felt that specialist knowledge was prioritized on objectives and examinations and was different than their practical day to day learning. UME was also understood to be interacting with elements of the macrosystem, such as accreditation, that learners felt were a driving force in some of their experiences, such as returning to the urban environment. While UME may be most appropriately placed in the exosystem, it clearly interacts with other exosystem components, like geographical isolation, as well as the microsystem, learning objectives and examinations, and the macrosystem, accreditation.

Learners highlighted disparities between the goals and resources of the urban environment and the realities of rural practice. Learning to work in an environment geographically distant from specialist care, and with limited resources was also highlighted as an important part of the exosystem. Learners understood a ‘real world’ in their rural communities, that stood in contrast to the well-resourced urban environment they experienced in pre-clerkship. This resource constrained ‘real world’ decision making, and the need for practical and patient centered thinking and decisions was felt to be key to working effectively in the rural setting, and while the disparities in healthcare access were noted, this was felt to be an important element of learning for students planning careers in the rural setting.

At the macrosystem level, learners had an awareness of the larger challenges of rural practice, such as workforce shortages, payment models, and burnout. Learners identified finances as being an important factor, but likely not a deciding factor in terms of future work and practice location. Resource disparities in the rural setting were readily identified by learners. Embedding social accountability objectives into the UME clerkship objectives and connecting these to the rural setting could support learning in this area and address the dissonance between specialist centered learning objectives and the reality of the rural setting.

Many of the learner responses across multiple layers of the model reflected the importance of the chronosystem. The LIC was not simply a set experience, but an experience that evolved over the learner’s time in the community. The relationship with the preceptor, integration into community, connection with other healthcare workers, as well as independence and development of professional identity were all elements that change and developed across the duration of the LIC. In the LIC setting continuity does not only refer to continuity with patients, or continuity with a primary preceptor, but also to continuity with a community and continuity as part of the healthcare team. This broad concept of continuity seemed to be an enabler of professional identity formation, with multiple learners describing how time in the community allowed them to understand themselves, take on progressive levels of responsibility, and achieve readiness for their next stage of training as residents.

Overall applying Bronfenbrenner’s ecological systems theory to the learning experience of the LIC reveals a layered, interconnected, and complex experience. Use of the model helps to unpack the LIC, expanding the range of recognized participants and interest holders in the process. While medical schools have been generally supportive of LICs, there may be an opportunity to better align exams and learning objectives more closely with the day-to-day experiences of rural generalist practice. Additionally, consideration should be given to engaging beyond the preceptors and healthcare workforce, facilitating a supportive learning environment within communities that allows learners to become a part of the community over their time in the LIC.

## 5 Strengths and limitations

The high participation rate of the LIC cohort is a key strength of the study. With a majority of LIC learners participating there is greater likelihood that the full range of viewpoints was captured in the study. The breadth of experience of the research team, including individuals highly involved with LIC as well as individuals more distant from LIC ensured a range of perspectives on the team and is also a strength.

There are also limitations of the study that may limit generalizability. This is a single institution study and a single cohort. The LIC learners were a mature group of students, many with prior professional careers, which may limit generalizability to medical students who are younger, with less prior professional experience. The cohort’s maturity and professional experience may have impacted their ability to interact broadly in the LIC setting and to form professional relationships beyond the primary preceptor relationship. Although we have tried to mitigate potential bias, the self-selected nature of the LIC cohort, and the rural medical education interests of the research team represent a group of people who likely have an overall positive outlook on rural medicine and rural medical education.

## 6 Conclusion

This qualitative analysis of the learning experience of a cohort of LIC learners revealed the complexity of the LIC learning environment. The centrality of the learner-preceptor relationship, highlighted in previous research, remains, but the importance of adjacent preceptors, the healthcare team, and integration into the community emerged as description of the entire learning experience. The importance of the primary preceptor as a connector to both the healthcare team and to the community deepened the understanding of the importance of this relationship. This analysis has broadened the concept of continuity in the LIC context, to also include community continuity alongside patient and preceptor continuity and emphasizes continuity as an enabler of professional identity formation in the LIC context.

The learners in this study demonstrated awareness of high-level issues including social and political factors impacting the healthcare environment and the challenges of UME administrative structures connecting to learners across significant geographic distances.

Bronfenbrenner’s model of ecological systems applied to an LIC environment allows a deeper understanding of the interplay between factors at different levels, and for the development of an LIC specific model, which may be of interest to LIC leaders and researchers.

## Data Availability

The datasets presented in this article are not readily available because of the nature of the placements. Only one or two LIC students are placed in the clinical sites, hence, the interactions and other situational experiences mentioned by the participants in the transcripts could make the individual personalities involved easily identifiable, despite applying great amounts of anonymization. Further inquiries can be directed to the corresponding author.
