# Supplementary material for: Becoming more integrated into the community: a qualitative study of learners’ experiences of the learning environment in a longitudinal integrated clerkship
Source: Front Med (Lausanne). 2025 Jul 11;12:1609051. doi: 10.3389/fmed.2025.1609051 (PMC12290413; doi:10.3389/fmed.2025.1609051)
Supplement: Supplementary file 1 [file Data_Sheet_1.PDF]

Appendix I - Interview Guide

I. Placement Information

1. Which rural community in Alberta were you placed?
   a. Which dates inclusive were you there?
   b. As applicable, did you bring your family (i.e., partner and children) with you?

II. Educational Experience

2. Overall, how satisfied are you with your rural immersion experience?
   a. What were the factors that contributed to your successful rural immersion?
   b. Community factors? Personal factors (e.g. physical, psychological, or social)?

3. How would you describe your exposure to continuity of care?
   a. Can you describe an experience of the natural progression of illness in a specific case or patient?
   b. Would you consider yourself prepared to handle undifferentiated patients?
   c. How has the rural immersion training helped you deal with uncertainty?

III. Community and Relationships Experience

4. What was your relationship with your preceptor like?
   a. How have you developed mutual trust?
   b. How would you describe teacher-learner relationship? Mentor-trainee? Friends?
   c. What teaching characteristics and other qualities did you find most useful in your learning?

5. How was your experience working with other professionals?
   a. What was it like to work with other allied healthcare professionals?
   b. What do you think of interprofessional care teams?
   c. What added value do you see in using interprofessional care team approach to patient care?

6. How was your experience living in the community?
   a. How involved were you in the community? (As applicable, how was your family?)
   b. How has living in the community affected your attitude toward rural practice?

7. What was your experience like being away from your family and friends during your placement?
   a. How did you manage feelings of isolation, if any?
   b. How did you stay connected?

IV. Career Plans

8. Would you say you received a comprehensive education?
   a. How has your rural immersion helped you adequately prepare for the exams?
   b. How confident do you feel for your next level of training (residency)?
   c. How would describe your knowledge of the Alberta Healthcare System before and after your rural immersion placement?

9. What were the results of your CaRMS match? Program name, university, location (rural or urban)?
   a. What was your first choice? Are you content with the match? Where else did you want to go?
   b. How has your rural integrated immersion helped you in the match?

V. Future Planning

10. How comfortable would you be living and practicing in a rural setting in the future?
11. Do you plan to practice in a rural community? If so, where?
12. What do you feel are areas of improvement for the longitudinal immersion of medical learners in the rural community?
